# Complete mitochondrial DNA sequence of oyster *Crassostrea hongkongensis*-a case of "Tandem duplication-random loss" for genome rearrangement in *Crassostrea*?

**DOI:** 10.1186/1471-2164-9-477

**Published:** 2008-10-11

**Authors:** Ziniu Yu, Zhengpeng Wei, Xiaoyu Kong, Wei Shi

**Affiliations:** 1 Laboratory of Marine Bio-resource Sustainable Utilization, Laboratory of Applied Marine Biology; South China Sea Institute of Oceanology, Chinese Academy of Sciences, 164 West Xingang Road, Guangzhou 510301, PR China; 2 Laboratory of Mariculture Research, Ocean University of China, 5 Yushan Road, Qingdao 266003, PR China

## Abstract

**Background:**

Mitochondrial DNA sequences are extensively used as genetic markers not only for studies of population or ecological genetics, but also for phylogenetic and evolutionary analyses. Complete mt-sequences can reveal information about gene order and its variation, as well as gene and genome evolution when sequences from multiple phyla are compared. Mitochondrial gene order is highly variable among mollusks, with bivalves exhibiting the most variability. Of the 41 complete mt genomes sequenced so far, 12 are from bivalves. We determined, in the current study, the complete mitochondrial DNA sequence of *Crassostrea hongkongensis*. We present here an analysis of features of its gene content and genome organization in comparison with two other *Crassostrea *species to assess the variation within bivalves and among main groups of mollusks.

**Results:**

The complete mitochondrial genome of *C. hongkongensis *was determined using long PCR and a primer walking sequencing strategy with genus-specific primers. The genome is 16,475 bp in length and contains 12 protein-coding genes (the *atp8 *gene is missing, as in most bivalves), 22 transfer tRNA genes (including a suppressor tRNA gene), and 2 ribosomal RNA genes, all of which appear to be transcribed from the same strand. A striking finding of this study is that a DNA segment containing four tRNA genes (*trnk1, trnC, trnQ1 *and *trnN*) and two duplicated or split rRNA gene (*rrnL5' *and *rrnS*) are absent from the genome, when compared with that of two other extant *Crassostrea *species, which is very likely a consequence of loss of a single genomic region present in ancestor of *C. hongkongensis*. It indicates this region seem to be a "hot spot" of genomic rearrangements over the *Crassostrea *mt-genomes. The arrangement of protein-coding genes in *C. hongkongensis *is identical to that of *Crassostrea gigas *and *Crassostrea virginica*, but higher amino acid sequence identities are shared between *C. hongkongensis *and *C. gigas *than between other pairs. There exists significant codon bias, favoring codons ending in A or T and against those ending with C. Pair analysis of genome rearrangements showed that the rearrangement distance is great between *C. gigas*-*C. hongkongensis *and *C. virginica*, indicating a high degree of rearrangements within *Crassostrea*. The determination of complete mt-genome of *C. hongkongensis *has yielded useful insight into features of gene order, variation, and evolution of *Crassostrea *and bivalve mt-genomes.

**Conclusion:**

The mt-genome of *C. hongkongensis *shares some similarity with, and interesting differences to, other *Crassostrea *species and bivalves. The absence of *trnC *and *trnN *genes and duplicated or split rRNA genes from the *C. hongkongensis *genome is a completely novel feature not previously reported in *Crassostrea *species. The phenomenon is likely due to the loss of a segment that is present in other *Crassostrea *species and was present in ancestor of *C. hongkongensis*, thus a case of "tandem duplication-random loss (TDRL)". The mt-genome and new feature presented here reveal and underline the high level variation of gene order and gene content in *Crassostrea *and bivalves, inspiring more research to gain understanding to mechanisms underlying gene and genome evolution in bivalves and mollusks.

## Background

As an organellar genome, animal mitochondrial DNA is typically a circular molecule of 15–20 kb, usually encoding 13 proteins, 22 transfer RNAs, and 2 ribosomal RNAs [1]. Thanks to its maternal inheritance, rapid evolutionary rate, and lack of recombination, fragments of mitochondrial DNA have been extensively used for studies of genetic structure, phylogenetics, and phylogeography at various taxonomic levels. Since studying complete mt sequences can uncover more information about gene order, rearrangements, and other variation at the genome level for all phyla, there have been significant increases in the number of complete mitochondrial sequences available in recent years [2-7]. It is known that mitochondrial gene order and its variation can be very useful for inferring evolutionary relationships [8]. Reportedly, molluscan species show an extraordinary amount of variation in gene arrangement, in contrast to the more limited gene rearrangement in many species of Arthropoda [3,4,6].

Of the 41 complete mollusk mt genomes available in GenBank, 12 of which are from bivalves, including *Mytilus edulis, Mytilus trossulus*, *Mytilus galloprovincialis*, *Venerupis philippinarum*, *Lampsilis ornata *etc., as well as two from oyster species. Oysters are distributed worldwide and are a species-rich bivalve group. Mitochondrial genomes of two oyster species, *C. gigas *and *C. virginica*, are available recently [5]. In comparing the mt-DNA of these two species, there is evidence of extensive genomic rearrangements and several duplications; mitochondrial genome information from additional species, therefore, would shed light on our relatively limited understanding of oyster evolutionary relationships.

*C. hongkongensis *(known as *C. rivularis*, previously) is primarily found in waters along the coast of the South China Sea [9]. With an annual landing of around 1.0 million metric tons, oyster farming of *C. hongkongensis *has been supporting one of the largest marine aquaculture industries in this area, marketing its products primarily to Hong Kong, Macau, Taiwan, and local markets as well. Populations were studied using different genetic markers, however, more polymorphic markers are needed for better and more detailed stock analysis [10-12]. With the process of urbanization and industrialization in some coastal regions, wild populations of this species have experienced some degree of decline. Meanwhile, the development of the oyster farming industry is driving resource management concerns and a desire for stock improvement. Stock enhancement is desired for stable and sustainable development of this industry. Interest in *C. hongkongensis *mitochondrial DNA has been increasing recently, partially due to the potential of mtDNA as a genetic marker for population analysis, stock management, and breeding programs. In this study, we determined the complete mitochondrial genome sequence of *C. hongkongensis*, in the hope of providing mt genome data for exploring possible mechanisms of gene rearrangements, addressing phylogenetic relationships among oysters and other molluscan species, as well as identifying more variable mtDNA regions for genetics studies and stock management of this important aquaculture resource.

## Results and discussion

### Genomic organization and structure

**Figure 1:**
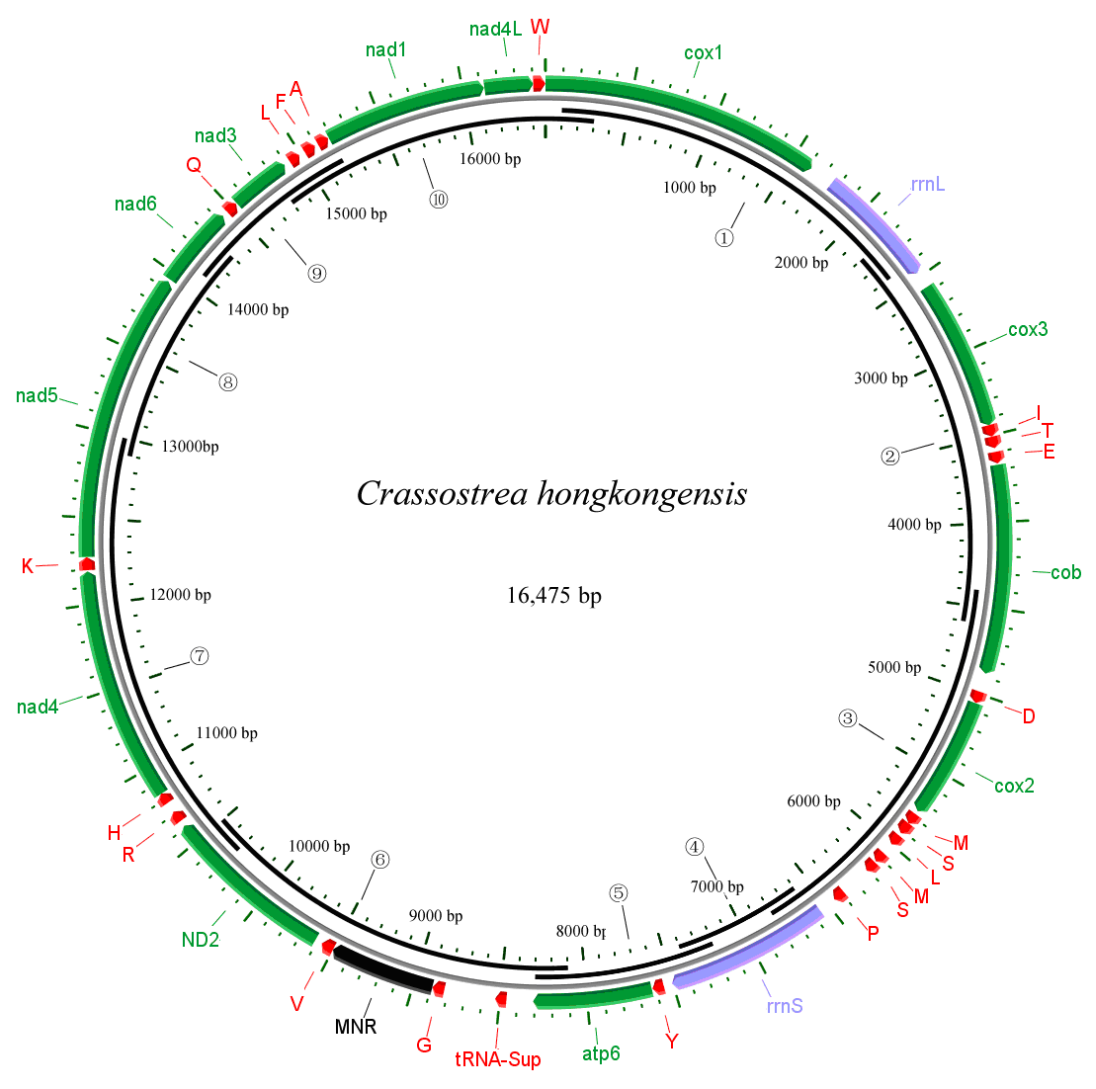
Gene map of the mitochondrial genome of *C. hongkongensis *and assembly indication of the ten overlapping large fragments (①~⑩) through PCR amplification.

**Figure 2:**
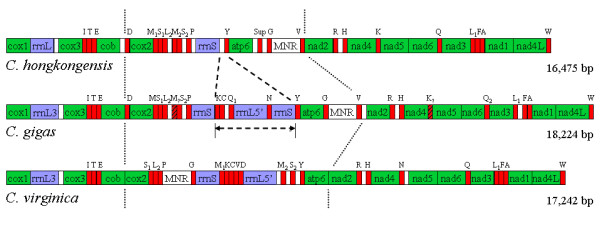
**Mitochondrial gene order and comparison of *C. hongkongensis*, *C. gigas *and *C. virginica*.** Genes are abbreviated as in the text. Noncoding regions are indicated by white boxes. Locations of *trnM*-like and *trnK*-like structure in *C. gigas *are hatched. Section between dotted lines is the region in which significant gene order rearrangements are present among the three oysters ("hot spots" of rearrangements). Segment between dash lines and arrows indicate the region present in *C. gigas *but absent in *C. hongkongensis*.

The mitochondrial genome of *C. hongkongensis *is 16,475 bp in length (GenBank accession number EU266073), shorter than that of the other two oysters whose mt-genomes have been sequenced, *C. gigas *(18,224 bp) and *C. virginica *(17,243 bp). However, the size of the *C. hongkongesis *mt-genome is certainly within the range of size of molluscan mtDNA genomes sequenced to date, i.e. from 13,670 bp in *Biomphalaria glabrata *to 32,115 bp in *Placopecten magellanicus*. The *C. hongkongensis *mtDNA contains 12 protein-coding genes (without *atp8*), 22 transfer tRNA genes (including a suppressor tRNA gene) and 2 ribosomal RNA genes (Fig. 1, 2 and Table 1), all apparently transcribed from the same strand, a common feature in marine bivalves.

**Table 1:** Features of *Crassostrea hongkongensis *mitochondrial genome

Feature	Sequence location	Size	Start codon	Stop codon	Intergenic region^§^
*cox1*	1–1620	1620	ATA	TAA	143
*rrnL*	1764–2475	712			96
*cox3*	2572–3441	870	ATA	TAA	0
*trnI*	3442–3508	67			0
*trnT*	3509–3576	68			21
*trnE*	3598–3665	68			7
*cob*	3673–4878	1206	ATA	TAA	8
*trnD*	4987–5055	69			1
*cox2*	5057–5758	702	ATG	TAG	21
*trnM1*	5780–5845	66			0
*trnS1*	5846–5917	72			16
*trnL2*	5934–6000	67			67
*trnM2*	6068–6132	65			7
*trnS2*	6140–6207	68			178
*trnP*	6386–6454	69			103
*rrnS*	6558–7505	948			49
*trnY*	7555–7620	66			5
*atp6*	7626–8309	684	ATG	TAG	152
*Sup*	8462–8527	66			294
*trnG*	8822–8891	70			0
*MNR*	8892–9499	608			0
*trnV*	9500–9572	73			42
*nad2*	9615–10613	999	ATG	TAG	34
*trnR*	10648–10714	67			60
*trnH*	10775–10839	65			-1
*nad4*	10839–12191	1353	ATA	TAG	7
*trnK*	12199–12273	75			1
*nad5*	12275–13945	1671	ATG	TAA	14
*nad6*	13960–14433	474	ATG	TAA	32
*trnQ*	14466–14534	69			5
*nad3*	14540–14890	351	ATG	TAG	35
*trnL1*	14926–14991	66			35
*trnF*	15027–15094	68			20
*trnA*	15115–15181	67			5
*nad1*	15187–16122	936	ATG	TAA	3
*nad4L*	16126–16405	280	ATG	T	0
*trnW*	16406–16474	69			1

^§ ^Intergenic region refer to noncoding bases between the feature on the same line and the feature on the line underneath, with a negative number indicating an overlap.

The arrangement of protein-coding genes in *C. hongkongensis *is identical to that of *C. gigas *and *C. virginica*, in the order of *cox1, cox3, cob, cox2, atp6, nad2, nad4, nad5, nad6, nad3, nad1 *and *nad4L *(Fig. 2), with tRNA genes punctuating the order [5]. However, the location of some tRNA genes, ribosomal RNA genes and a major noncoding region (MNR) differ to a great extent among the three *Crassostrea *genomes (Fig. 2). The overall genomic organization of *C. hongkongensis *is more similar to that of *C. gigas *than to *C. virginica*, corresponding evidently to their closer genetic relationship. In contrast, in *Mytilus *congeners *M. edulis, M. trossulus *and *M. galloprovincialis*, not only are protein-coding genes arranged in the following identical order: *cox1, atp6, nad4L, nad5, nad6, cob, cox2, nad1*, *nad4, cox3, nad2 *and *nad3*; but also the order of tRNAs, ribosomal RNA, and control region are almost the same. In other words, gene arrangement is highly conserved among *Mytilus *species.

The major mt-genomic region significantly different among the three *Crassostrea *genomes is that between *cob *and *nad2 *(Fig. 2), with other regions being almost identical. In this variable region, *C. gigas *and *C. virginica *have a segment (between *rrnS *and *trnY*) that is completely absent in *C. hongkongensis*. This is a totally striking finding regarding mt genome structure of *Crassostrea*. The segment contains split *rrnL5'*, duplicated *rrnS *and four tRNA genes in *C. gigas*, and split *rrnL5' *and seven tRNA genes in *C. virginica*, respectively. Additionally, there is obvious variation of gene order in the *C. virginica *when compared with the other two species: MNR is translocated between *cox2 *and *rrnS*, and some tRNA genes are rearranged. Therefore, this section seems to be an obvious "hot spot" of rearrangement in *Crassostrea *mt genome.

The relationship of genome length in the 3 *Crassostrea *species may be a reflection of the segment's loss: although *C. gigas *is more closely-related to *C. hongkongensis *than to *C. virginica*, the genome length difference between them (1749 bp) is greater than that between *C. gigas *and *C. virginica *(982 bp). Similarly, the lack of duplicated *rrnS *in *C. virginica *in this segment may account for the length difference between *C. gigas *and the *C. virginica*. As observed by Rawlings et al. in *Dendropoma*, this kind of variation in gene order within a genus is likely to be associated with "hot spots" of rearrangements, and may be explained by an intra-mt recombination model [13].

Although gene overlap is common in animal mt genomes, there are only two such genes in *C. hongkongensis *mt genome, *nad4 *overlaps with *trnH *by 1 nucleotide (Table 1), while in *C. gigas *and *C. virginica *there are no overlapping genes [14]. Additionally, four protein-coding genes directly abut each other as they do in *C. gigas*:*nad5-nad6 *and *nad1-nad4L*. In *C. virginica*, 8 genes abut each other: *cob-cox2 *and *atp6-nad2*, in addition to the two pairs just mentioned [5]. As usual, no introns were found, thus it is likely that all of the genes from the coding strand are expressed as a polycistronic RNA that is then processed enzymatically to release the protein-coding genes' mRNAs, as reported in a few cases [15]. Since tRNA secondary structures play a crucial role in RNA maturation from the polycistronic transcripts, it is not clear by what mechanism transcript cleavage would occur at the 3' end of *nad5, nad1 *and *cox1*, as these are not flanked by tRNA genes [16].

Noncoding regions between genes totaled to 1,561 bp in length, with 608 bp in the MNR and another 953 bp dispersed in 29 intergenic regions (Table 1). The A+T composition of *C. hongkongensis *mt-DNA is 65.4%, lower than that of many other invertebrates, but comparable to the 63.3% A/T composition of *C. gigas *mt-DNA, as well as that of *C. virginica *(61.1%) and *M. edulis *(61.8%). This A+T bias pattern holds for all protein-coding, tRNA, rRNA genes while noncoding nucleotides except MNR, display an even higher A+T content of 77.8%, significantly higher than the 65.8% seen in *C. virginica *and 69.5% seen in the Pacific oyster. Strand skew measures for the distribution of base pairs show an AT skew [(T-A)/(T+A)] value of 0.134 and GC skew [(G-C)/(G+C)] value of 0.207, respectively, almost the same as those of *C. gigas *and very similar to those of *C. virginica *[5,17]. This indicates that the strand containing genes is quite rich in T and G in contrast to that of other mollusks like *Graptacme eborea*, in which the skew values are extremely close to zero [4]. The observed G and T richness of the gene coding strand is evidence of codon usage bias.

### Transfer RNA genes

In total, 21 tRNA genes plus a putative suppressor tRNA gene were identified based on their respective anticodons. Sequences complementary to the coding strand (including that for the suppressor tRNA) can form an expected cloverleaf structure, with ranging in size from 65 to 75 nucleotides. As found in the other two *Crassostrea *mt-sequences as well as in mtDNA of some other species (*Katharina tunicata*, *Cepaea nemoralis*, *M. edulis, M. galloprovincialis, Argopecten irradians*), two serine and two leucine tRNA genes are differentiated in *C. hongkongensis *by their anticodons (UUA for Leu1, CUA for Leu2, AGA for Ser1, UCA for Ser2). Similarly, an additional *trnM *with a cognate anticodon was also detected, as found in *C. gigas*, *C. virginica, V. philippinarum *and *Mytilus*.

The new finding in this study is that *trnC *and *trnN *genes are absent from the mt genome of *C. hongkongensis *(Fig. 2), a phenomenon not reported before for any other mollusk species with mitochondrial genome data available so far, including *Mizuhopecten yessoensis*, which only has nine identified tRNA genes, though. To confirm the absence of these genes, the mt genome of one more individual of *C. hongkongensis *was sequenced. Both tRNA genes (and duplicated or split ribosomal gene) were still found absent in the second individual as well. Although tRNAs play a crucial role in RNA maturation from polycistronic transcripts, the number of distinct tRNA genes present in mt genomes varies greatly across eukaryotes. Reportedly, the loss of a tRNA gene (*trnE*) was detected also in Antarctic fish and it co-occurred with the loss of *nad6 *gene [18]. It is predominantly thought that the loss of tRNA genes is ameliorated via import of nuclear tRNAs [19]. Comparison of gene order (including tRNA genes) between *C. hongkongensis *and *C. gigas *shows that the two genomes are almost identical, except for the segment between *rrnS *(the one abutting *trnP*) and the *trnY *gene in *C. gigas*, which (the segment) is absent from *C. hongkongensis *(Fig. 2). This segment of *C. gigas *contains *rrnL'5*, duplicated *rrnS*, and tRNA genes *trnK, trnQ1*, *trnC *and *trnN*; all of these loci are absent in *C. hongkongensis *(thus *C. gigas *has 4 more tRNA genes than does *C. hongkongensis*). The absence of the four tRNA genes and two duplicated or split rRNA genes could be explained reasonably if we suppose that the ancestor of *C. hongkongensis *had this segment as *C. gigas *currently does and then it was lost through DNA rearrangement. This is very likely an example of "tandem duplication-random loss (TDRL)" mechanism for gene rearrangement. Meanwhile, since extra copies of other two tRNA gene *trnK *and *trnQ *are found outside this region, their loss does not lead to a complete absence of these two tRNA genes from the mitochondrial genome.

Interestingly, a putative suppressor tRNA gene was identified. This suppressor tRNA has an anticodon sequence of 3'AUU, corresponding to nucleotides 8462–8527 of the complete DNA sequence. It therefore is a nonsense suppressor, and could recognize the stop codon UAA in the mRNA and, instead of terminating, insert an amino acid at that position in the polypeptide chain [20]. A BLAST search failed to identify any homologous sequence of the putative suppressor tRNA gene, although the tRNA has a predicted secondary structure similar to that of other tRNAs. Lastly, as seen in *C. gigas*, *C. virginica *and many other mollusk species, the Leu2 (UUA) tRNA gene contains the mitochondrial rRNA termination box, a quite conserved heptamer TGGCAGA, at nucleotides 8 to 14 [21].

### Protein-coding genes

Among the supposed 13 protein-coding genes, 12 were identified in *C. hongkongensis *through open reading frame (ORFs) searching. No *atp8 *coding sequence was detected in this process. Boore [1] mentions that a lack of the *atp8 *gene is one of several unusual features of the *Mytilus *mt sequence. The *apt8 *gene is missing from the mt-DNA of almost all bivalve species studied so far, including *C. gigas*, *C. virginica, Mytilus, P. magellanicus, A. irradians, M. yessoensis *and *Acanthocardia tuberculata*. The one exception found so far is *Hiatella arctica *in which the *apt8 *gene is present. Interestingly, *V. philippinarum*, originally not annotated for gene *atp8 *in its mt-genome, was recently found to contain a putative *atp8 *gene, though it apparently only encodes 37 amino acids and therefore has questionable gene function [7]. In contrast, all Gastropoda species (14) studied to date possess an *atp8 *gene, and as do all Cephalopoda species (11) examined up to now. Other mollusk species, from Polyplacophora *K. tunicata *to Scaphopoda *G. eborea *and *Siphonodentalium lobatum*, have an *atp8 *gene as well.

The 12 genes in *C. hongkongensis *are similar in length to their counterparts in *Crassostrea*. However, *C. hongkongensis *and *C. gigas *share a higher degree of amino acid similarity in 8 genes than do *C. hongkongensis *or *C. gigas *and *C. virginica*. Also, a higher level of amino acid identity is shared between *C. hongkongensis *and *C. gigas *than that is seen between other pairs (Table 2). All protein-coding genes start with typical invertebrate initiation codons, with 8 employing ATG and the other 4 using ATA. Six protein-coding genes were terminated by a TAA and five by a TAG codon, and one by an incomplete termination codon T--, with its likely completion occurring by polyadenylation after transcript processing [15].

**Table 2:** Protein-coding gene assignments and identity of the three *Crassostrea *species

	Number of amino acids			% identity			% identity in congeners	
Protein	Ch*	Cg*	Cv*	*Ch-Cg*	*Ch-Cv*	*Cg-Cv*	*Crassostrea*^§^	*Mytilus*^†^
*cox1*	539	538	540	97.6	92.8	92.0	91.7	98.9
*cox3*	289	291	290	87.8	63.3	64.0	61.9	83.0
*cob*	401	412	403	85.5	71.3	71.8	68.5	84.6
*cox2*	233	233	230	98.7	91.0	91.0	90.6	97.5
*atp6*	227	227	224	96.9	70.2	70.6	69.7	97.5
*nad2*	332	332	331	87.7	49.1	47.0	57.5	89.9
*nad4*	450	450	449	81.4	71.2	67.8	63.9	92.0
*nad5*	556	556	555	79.2	62.9	60.0	56.6	91.4
*nad6*	157	158	153	77.2	57.6	54.4	51.3	66.5
*nad3*	116	116	117	87.1	67.5	61.5	60.7	95.7
*nad1*	311	311	311	87.8	73.6	70.7	69.1	96.7
*nad4L*	93	94	93	91.5	74.2	68.1	67.0	98.9

*Ch: *Crassostrea hongkongensis*, *Cg*: *C. gigas*, *Cv*: *C. virginica*; ^§ ^refer to the identity among the 3 *Crassostrea *species: *C. hongkongensis*, *C. gigas *and *C. virginica*; ^† ^refer to the identity among the 3 *Mytilus *species: *M. edulis, M. trossulus *and *M. galloprovincialis*.

Amino acid identity in proteins for oyster pairs ranged from as low as 47% between *Cg-Cv *in *nad2 *to as high as 98.7% between *Ch-Cg *in *cox2*. The overall level of the identity among the three *Crassostrea *species varied from 51.3% (*nad6*) to 91.7% (*cox1*), significantly lower than the corresponding values among three congeneric *Mytilus *species. Just as the gene order is less conserved than that in *Mytilus *species, *Crassostrea *species exhibit lower conservation of amino acid identity in all protein-coding genes (Table 2). Amino acid identity is also much lower than that found amongst five *Drosophila *species [5]. According to our data (Table 2), it appears that the most conserved protein-coding genes are *cox1 *and *cox2 *(identity > 90%), and the least conserved are *nad2*, *nad5 *and *nad6 *(identity < 60%, Table 2) in *Crassostrea*.

### Gene order and pair analysis of genome rearrangements in Crassostrea

It's well known that gene arrangements usually remain steady over long periods of evolutionary time (especially for protein-coding genes), in contrast to the significant variation and rapid evolution of mtDNA sequences [1]. With some exceptions, mitochondrial gene order is relatively stable within major groups, and generally variable among groups [22]. However, this appears not always to be the case in mollusks. While mt-gene rearrangements appear to be extensive in the major groups of mollusks, Rawlings et al. reported that protein-coding gene rearrangements have occurred even within a genus (e.g. in the vermetid gastropod *Dendropoma*), indicating that dramatic changes could also take place at the level of fine-scale phylogeny [13]. Several possible mechanisms have been proposed to explain gene rearrangements in mt genomes, including TDRL and "intra-mt recombination" models. TDRL is believed to occur primarily in vertebrates, but it is likely to occur in mollusks as well, as exemplified putatively by the occurrence of the duplicated gene block *cox1-cox2-atp8-atp6 *and *cox3 *as an intermediate state in this process for *Dosidicus gigas *and *Todarodes pacificus *in Cephalopoda, and also by the absence of the segment of tRNAs-duplicated rRNA genes found in this study as a consequence in the proceeding for *C. hongkongensis *[23,24]. Furthermore, the occurrence and subsequent differentiation of duplicated tRNA genes and rRNA genes in some species (such as *C. gigas *and *C. virginica*) may be another consequence following this rearrangement. Since this model cannot explain rearrangements in which a gene moves from one strand to the other, intra-mt recombination is thought to account for both these rearrangements as well as gene loss. Theoretically, when two proximate double-stranded breaks occur and the DNA circularizes to form mini-circles, segment loss often occurs; if the circular piece is re-inserted into the genome, inversion (reversal) is easily produced [25,26]. These seem to occur frequently in mollusks across groups and species.

According to available data, bivalves apparently have significantly great amount of mt gene rearrangement, with gene translocation across all gene classes and very few shared gene boundaries. On the other hand, loss of protein-coding genes is also a common phenomenon, as mentioned above. It is known that some mt protein-coding genes were gradually lost, functionally and then physically, over long evolutionary periods of time. Physical loss of the *atp8 *gene, for example, was first detected in nematodes, and then in *Mytilus*, *Crassostrea*, *M. yessoensis *and male mitotype of *V. philippinarum*, although a remnant of its ancestral gene was detected in *C. virginica *[3,5,27-29]. Functional loss of *atp8 *was revealed in female mitotype of *V. philippinarum*. In Antarctic fish, even the *nad6 *gene was also lost [18]. Based on the situation mentioned above, it could be possible, as more data become available, to find additional bivalve species lacking *atp8 *or other genes, provided these genes are replaced by functional gene transfer to the nucleus [30].

**Figure 3:**
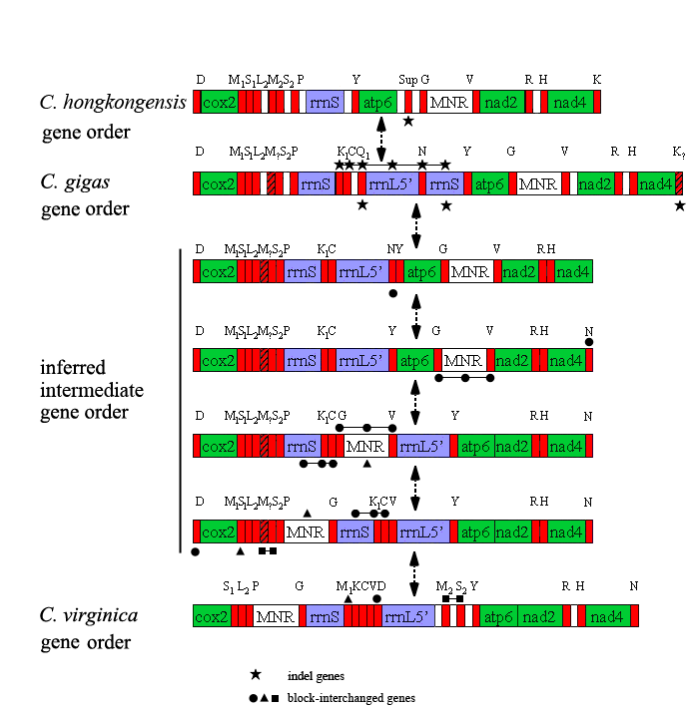
**Pair analysis of mitochondrial genome rearrangements with SPRING (Sorting Permutation by Reversals and block-INterchanGes) in the three *Crassostrea *species.** Star symbol denotes indel genes; dots, solid triangles and squares indicate block-interchange genes during putative genome rearrangements of *Crassostrea*, respectively. Symbols connected by line denote abutted genes. Double-arrows represent directions and steps in putative procedure of genome rearrangements.

Genome rearrangement studies are based on genome-wide analysis of gene orders. The variation of mt gene order occurred in *Cassostrea *were examined closely through pair analysis of genome rearrangements and direct comparison. It is shown that there are at least five permutations between *C. gigas *and *C. virginica *(Fig. 3): indel of *trnQ1*, *trnK *and duplicated rrnS; transposition of *trnN*; transpositions of *trnG, trnV *andMNR; transpositions of *trnK1, trnC*, rrnS and MNR; and transpositions of *trnD, trnM1, trnM2 *and *trnS2*. However, only one single permutation is inferred between *C. gigas *and *C. hongkongensis*, involving the indel of four tRNA genes (*trnK1, trnC, trnQ1 *and *trnN*) and two duplicated or split rRNA genes (*rrnL5' *and *rrnS*), obviously. With three *Cassostrea *mtDNA genomes, it was supposed to be able to find ancestral genome scenario. However, the distinct feature (absence of a DNA region) of *C. hongkongensis *mtDNA genome prevented the analysis of reconstructing rearrangement: only the genes that all genomes involved have in common are considered for analysis, i.e. repetitions or gaps (indels) in genomes are excluded. As gene order of *C. hongkongensis *and *C. gigas *would be the same if the indel between them is excluded, pair analysis of genome rearrangements in the three genomes would be actually conducted for two genomes (Fig. 3) and hence no ancestral genome scenario could be found for *Cassostrea*. Clearly, the rearrangement distance of 5 between *C. gigas *and *C. virginica *is a great value within a genus, indicating a high degree of rearrangements. Obviously, the distance between *C. hongkongensis *and *C. gigas *would normally be 1, even without analysis.

The commonly occurred rearrangements are reversals and block-interchanges (generalized transpositions). However, the latter should be the dominant one in bivalves studied to date, because all these genomes have a single transcriptional orientation, except for *L. ornata*. Clearly, the absolute majority of block-interchanges must have occurred in the "hot spot" region for the 3 *Cassostrea *genoms (Fig. 3). That if this is true in other *Cassostrea *species could be verified when more mt DNA genomes become available in the future.

### Codon usages and codon bias

A total of 3704 amino acids are encoded by the *C. hongkongensis *mitochondrial genome, compared with 3718 and 3696 for its counterpart *C. gigas *and *C. virginica*, respectively. Similar to these two counterparts, leucine (468) and serine (420) are the most frequently encoded amino acids, followed by valine (334) and phenylalanine (308); arginine (59) is the least frequent. Individually, UUU (Phenylalanine) is definitely the most frequently used codon not only in *C. hongkongensis *(7.7%), but also in *C. gigas *(7.4%) and *C. virginica *(5.8%) as well, followed by AUU (Isoleucine) in the three species (5.7%, 5.8% and 5.2%, respectively).

**Figure 4:**
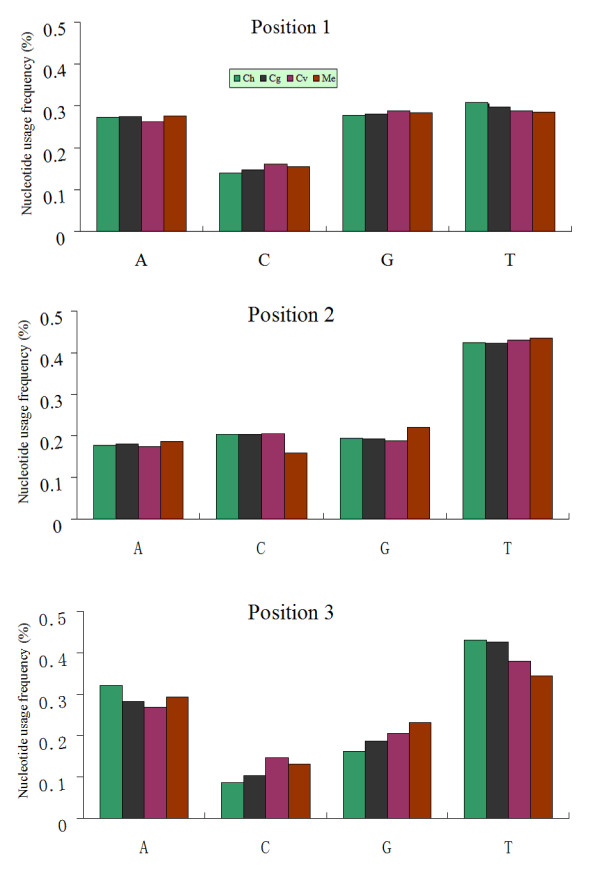
**Nucleotide usage frequency of three *Crassostrea *species with *Mytilus edulis *as a reference.** Ch: *Crassostrea hongkongensis*, Cg: *C. gigas*, Cv: *C. virginica *and Me: *M. edulis*.

Moreover, among 3704 codons in *C. hongkongensis*, 2788 (75.3%) end in an A or T, a more pronounced percentage than that observed in the other two *Crassostrea *species (71.0% and 64.7%, respectively) but a phenomenon observed in the typical invertebrate codon bias. There is a strong bias against the use of C (8.6%) at the third position nucleotide in all codons. In detail, for amino acids with a fourfold degenerate third position, codon families ending with T are the most frequently used, except in serine1 and arginine codons. Codons ending with A are used next most frequently. This is also the case for twofold degenerate codons. In other words, in every case where an amino acid can be specified by any NNY codon, the *C. hongkongensis *has a much higher proportion of NNT: NNC. At the second position, there is even a stronger bias in favor of the use of T (42.5%), which is also true for *C. gigas *(42.3%), *C. virginica *(43.0%) and *M. edulis *(43.5%). While at the first codon position, T (30.9%) has the highest percentage followed by G (27.8%), so are the cases in species mentioned above (Fig. 4).

Leucine can be specified by six different codons of TTR and CTN, and the proteins of the *Crassostrea *species have a very similar number of leucines (468, 461 and 472, respectively). As the reflection of base bias in codons, *C. hongkongensis*, together with *C. gigas *and *C. virginica*, has a significantly greater percentage of leucines encoded by TTA and CTT codons (with range of 53.2–67.0%), primarily at the expense of CTC and CTG codons. Similarly, in the case of serine with 8 different codons (TCN and AGN), a much higher proportion (with range of 60.1–65.5%) of AT rich codons of TCT, AGA and TCA are clearly observed in *Crassostrea *congeners.

### Ribosomal RNA genes

BLAST searches assigned locations of the 12S and 16S rRNA genes in the *C. hongkongensis *mt-genome. The 12S rDNA is contained in a 948 bp region (6558–7505) flanked by *trnP *and *trnY*, and as seen in *C. virginica*, but in contrast to *C. gigas*, no duplicated small subunit rRNA gene was detected. Alignment of the 12S rDNA from the three oysters shows that there is a fairly homologous core of 979 nucleotides at 78.3% identity (with 37 nucleotide indels and 175 substitutions), corresponding to 6558–7505 for *C. hongkongensis*, 3855–4800 and 5931–6879 (duplicated) for *C. gigas*, and 6847–7797 for *C. virginica*, respectively. However, if only *C. hongkongensis *and *C. gigas *are considered, the homologous core is 949 nucleotides long with 96.7% identity, with two indels and 29 substitutions, corresponding to 6558–7505, and 3855–4800 and 5931–6879 (duplicated) in the genomes, respectively.

The 16S rRNA gene is 712 bp long, flanked by protein-coding genes *cox1 *and *cox3*. Unlike the 16S rRNA gene in *C. gigas *and *C. virginica *mt-DNA, it is not split or duplicated into two fragments (Fig. 2) [5]. The 16S rRNA gene of *C. hongkongensis *and *rrnL3' *half of *C. gigas *shows nucleotide identity of 95.8% over 715 aligned bases (including 3 indels and 27 substitutions), while it is 82.4% between *C. hongkongensis *and *C. virginica *over 721 nucleotide alignment (including 11 indels and 116 substitutions). Unlike the high identity of *rrnS *among three *Crassostrea *(including the duplicated one in *C. gigas*), there is low identity between *rrnL3 *and *rrnL5 *(36.4%) in *C. gigas*, perhaps a consequence of *rrnL*'s modest evolutionary fragmentation, rather than duplication [31].

### Noncoding regions

As in other two oysters and *Mytilus*, the *C. hongkongensis *mt genome includes a large number of noncoding nucleotides, in contrast to *G. eborea*. Excluding the 3'UTR sequences for protein-coding genes, there exist large intergenic segments in *C. hongkongensis *and 10 of which were larger than 50 bp in length. Among these regions, a 608 nucleotide segment between *trnG *and *trnV *was putatively identified as the major noncoding region (MNR; Table 1, Fig. 1 and 2), on the basis of its noncoding characteristics and A+T richness (77.8%), a feature typically used for identification of the mitochondrial control region and thought to contain replication origin [1]. Additionally, several (A)_n _and (T)_n _homopolymer tracts were contained in this region. The second largest intergenic sequence was a fragment between suppressor tRNA gene and *trnG*, with a length of 294 bp and A+T content of 70.8%. Alignment of MNR between *C. hongkongensis *and *C. gigas *detected a fragment of 60 bp long, with 90.0% sequence identity and A+T content of 83.3% and 80.0%, respectively, or a sequence of 51 bp in length, with 96.1% identity, A+T content of 80.4% and 82.4%, respectively.

## Conclusion

Although the arrangement of protein-coding genes of *C. hongkongensis*'s mt genome is identical with that of *C. gigas *and *C. virginica*, and a moderate to high level of gene/amino acid identity is shared among the three *Crassostrea *species, *C. hongkongensis *exhibits a high degree of variation in gene order and gene content. The most striking of these are the absence of the two tRNA gene *trnC *and *trnN *and duplicated or split rRNA genes. Based on a comparative analysis, we assume that the absence of these genes is the result of evolutionarily loss of a genomic segment that was present in the ancestor of *Crassostrea*, and a likely case of "Tandem duplication-random loss" for genome rearrangement. While this novel and interesting feature of *C. hongkongensis *and the comparison of mt-genomes among the three *Crassostrea *species presented here have yielded useful insights into possible mechanisms underlying variation of gene order and gene content change for *Crassostrea*, more information could be expected from mt-genome studies of other oysters in Ostridae, promising intensive understanding of gene order/content change, as well as tRNA mutation and genome evolution for oysters and other bivalves.

## Methods

### PCR and sequencing

Adductor muscle from a *C. hongkongensis *individual collected in Beihai, Guangxi province, China was used for this study. Total genomic DNA was extracted using a standard phenol/chloroform method [32]. Based on alignment and comparison of complete mitochondrial genome sequences of *C. gigas *and *C. virginica*, ten genus-specific primer pairs were designed for amplifications of mtDNA large fragments in *C. hongkongensis *(Table 3), and then the complete mitochondrial genome was amplified in 10 overlapping large fragments accordingly (Fig. 1). PCR was performed in 25-μl reaction volume, containing 2.0 mM MgCl_2_, 0.2 mM dNTP, 0.5 μM of each primer, 1.0 U Taq polymerase, 1× PCR buffer and 1μl template DNA. PCR cycling condition were 94°C for 2 min; then 35 cycles of 94° for 1 min, annealing temperature for 1 min and 72°C for 1.5 min; with a final step of 72°C for 5 min. PCR products were checked by electrophoresis on 1% agarose gel and purified using Qiagen PCR Purification kits (Qiagen, USA). Purified products were then used as templates directly for cycle sequencing reactions. Species-specific primers were designed and used for primer walking sequencing, which was performed for both strands of each sample on an ABI 3730 DNA sequencer (ABI, USA). When two mt tRNA genes was found absent in the individual of *C. hongkongensis *sampled, the genome for one more individual was then sequenced for confirmation.

**Table 3:** Primers used for amplification of large fragments in *Crassostrea. hongkongensis *mitochondrial genome

Order	Primer name	Sequence (5'-3')	Amplification conditions*	Product size (bp)
1	RCOIA Mt133	GGTCAACAAATCATAAAGATATTGG CCGGTCTGAACTCAAATCA	35× (94°C 1 min, 57°C for 1.5 min, 72°C for 1.5 min)	2325
2	Mt89 1R	CAGTACCTGCCCAGTGCGACAT GGCTTAATTACGGCTGGTGTTT	35× (94°C 1 min, 57.5°C for 1.5 min, 72°C for .5 min)	2626
3	CYF 12-B	TTAGAGTTCCGTTTCACCCG CTTTCGCTGCGGTTTAGTTAGT	35× (94°C 1 min, 47.5°C for 1 min, 72°C for 1.5 min)	2448
4	12-F 12B	GGTTCTGGTCTAATGTTCGCT GTTACTCTCCCTTTACTCCC	35× (94°C 1 min, 47°C for 1 min, 72°C for 1 min)	804
5	12RF ATP-H	GTAGGTCAGGACGAAGTGCT AGAGCACAGGTGTTGGGAGA	35× (94°C 1 min, 49°C for 1 min, 72°C for 1 min)	996
6	727F DNR	TATTCGCCCTGACACTCTTAC TCCCGTATTCCAGAAGAAGCAG	35× (94°C 1 min, 77.5°C for 1 min, 72°C for 1.5 min)	2444
7	ND2F 5NR	AGATTTGCTGGTTTTCCTCCG CTGATGTTGTAAGTCCCGCAC	35× (94°C 1 min, 49.5°C for 1 min, 72°C for 1.5 min)	2641
8	ND6F2 NDR	GCTCCTACTCCTGTCTCATC TGACGCTTGCGAATAAGACAC	35× (94°C 1 min, 49°C for 1 min, 72°C for 1 min)	948
9	ND5F RND	GAGGATTTACTGCGGTGATTGG GTCAGAGCCATTCCGACTATC	35× (94°C 1 min, 47.5°C for 1 min, 72°C for 1.5 min)	1373
10	FLeu NO1	GCCAGGTTAGTGTGGTATTTAG GAGTAAGTGGATAAGGGTGGA	35× (94°C 1 min, 47.5°C for 1 min, 72°C for 1.5 min)	2017

* Steps of initial 94°C for 2 min at the beginning and final 72°C for 5 min after 35 cycles are omitted in cycling profile of each primer pair in the table.

### Sequence analysis and gene order comparison

During the processing of large fragments and those from walking sequencing, regular and manual examinations were used to ensure reliable overlapping and correct genome assembly. Protein-coding and ribosomal RNA genes were firstly identified using BLAST searches at GenBank, and then by alignment with previously published mt genomes from species of *Crassostrea*, *Mytilus *and other closely related mollusks [33]. Amino acid sequences of protein-coding genes were inferred with ORF Finder using invertebrate mitochondrial genetic code [34]. Identification of tRNA genes was conducted with tRNAscan-SE using mito/chloroplast genetic code and default search mode or setting the cove cutoff score to 1 when necessary [35,36]. Potential cloverleaf structures for identified tRNAs were determined in tRNAscan-SE. Comparisons of mitochondrial gene order were conducted and facilitated with published mollusk genomes from GenBank and OGRe (the Organellar Genome Retrieval system) web site [37-39]. Pair analysis of genome rearrangements was made with SPRING (Sorting Permutation by Reversals and block-INterchanGes) [40,41]. GenBank accession numbers of mt genomes from the taxa used in this study were listed in Table 4. Gene map of the mitochondrial genome of *C. hongkongensis *was generated using CGView [42].

**Table 4:** List of taxa used for genome comparison

Taxon and Species	Accession No	Taxon and Species	Accession No
Bivalvia		*Venerupis philippinarum*	NC_003354
*Acanthocardia tuberculata*	NC_008452	Gastropoda	
*Argopecten irradians*	EU023915	*Biomphalaria glabrata*	NC_005439
*Crassostrea gigas*	NC_001276	*Cepaea nemoralis*	NC_001816
*Crassostrea virginica*	AY905542	Cephalopoda	
*Crassostrea hongkongensis*	EU266073	*Dosidicus gigas*	NC_009734
*Hiatella arctica*	NC_008451	*Todarodes pacificus*	NC_006354
*Lampsilis ornate*	AY365193	Polyplacophora	
*Mizuhopecten yessoensis*	NC_009081	*Katharina tunicate*	NC_001636
*Mytilus edulis*	AY484747	Scaphopoda	
*Mytilus galloprovincialis*	NC_006886	*Graptacme eborea*	NC_006162
*Mytilus trossulus*	NC_007687	*Siphonodentalium lobatum*	NC_005840
*Placopecten magellanicus*	NC_007234		

## Authors' contributions

ZY designed the research and performed most of the data analyses; he also conducted examination of initial annotation and re-annotation, drafted and finalized the manuscript. ZW carried out most of the experiments (including PCR, sequence check and assembly) and initial annotation; XK initiated, led the research, and supervised all laboratory work; WS participated in data analyses and made all figures.
